# Macrophage energy metabolism in cardiometabolic disease

**DOI:** 10.1007/s11010-024-05099-6

**Published:** 2024-08-29

**Authors:** Angela Wong, Qiuyu Sun, Ismail I. Latif, Qutuba G. Karwi

**Affiliations:** 1 Division of BioMedical Sciences, Faculty of Medicine, Memorial University of Newfoundland, St. John’s, Newfoundland and Labrador A1B 3V6 Canada; 2 Cardiovascular Research Centre, University of Alberta, Edmonton, Alberta Canada; 3 Department of Microbiology, College of Medicine, University of Diyala, Baqubaa, Diyala Iraq

**Keywords:** Macrophage, Metabolism, Heart failure, Obesity, Diabetes

## Abstract

In a rapidly expanding body of literature, the major role of energy metabolism in determining the response and polarization status of macrophages has been examined, and it is currently a very active area of research. The metabolic flux through different metabolic pathways in the macrophage is interconnected and complex and could influence the polarization of macrophages. Earlier studies suggested glucose flux through cytosolic glycolysis is a prerequisite to trigger the pro-inflammatory phenotypes of macrophages while proposing that fatty acid oxidation is essential to support anti-inflammatory responses by macrophages. However, recent studies have shown that this understanding is oversimplified and that the metabolic control of macrophage polarization is highly complex and not fully defined yet. In this review, we systematically reviewed and summarized the literature regarding the role of energy metabolism in controlling macrophage activity and how that might be altered in cardiometabolic diseases, namely heart failure, obesity, and diabetes. We critically appraised the experimental studies and methodologies in the published studies. We also highlighted the challenging concepts in macrophage metabolism and identified several research questions yet to be addressed in future investigations.

## Introduction

The innate immune system is the primary and most effective line of defense against infectious pathogens. It comprises various innate immune cells, such as macrophages, monocytes, granulocytes, and dendritic cells. These cells recognize pathogen-associated and damage-associated molecular patterns (PAMPs and DAMPs) through pattern recognition receptors (PRRs) like Toll-like receptors (TLRs), advanced glycation end products (RAGE), and Nod-like receptors (NLRs) [1, 2]. This recognition triggers multiple signaling pathways that produce reactive oxygen and nitrogen-related species, chemokines, pro-inflammatory cytokines, and antimicrobial-associated peptides [1, 3]. These molecules efficiently eliminate microbial infections. Furthermore, the innate immune system activation triggers more specific adaptive immunity [4–6]. However, the innate immune system can also cause inflammation that can harm the host. Therefore, it is crucial to understand the immune regulatory strategies that control the initiation, extent, and goals of the inflammatory process.

Macrophages are a type of immune cell discovered by Elia Metchnikoff in 1882 [4]. He found them in starfish hatchlings, where they were responsible for the phagocytosis of foreign materials using tangerine tree thistles. Later, he found them in Daphnia magna or essential water fleas infested with fungal spores [7, 8]. Macrophages are an important part of our innate immune system and are crucial in regulating several homeostatic and evolutionary host defense immune responses. They also have several other functions, such as regulating reactive oxygen species (ROS) levels, managing iron levels, repairing tissue injury, and performing many other metabolic functions. Additionally, macrophages have three essential functions: they modulate the immune system, phagocytose foreign materials, and present antigens. They are vital for normal immune reactions in various pathophysiological conditions.

Macrophages also play indispensable roles in tissue repair and maintaining the body’s overall balance [4]. Despite their significance, we still have limited knowledge about the signal transduction processes within these cells to mediate these functions. Also, the immune pathways affected during healthy and diseased states are not fully understood. It has long been recognized that an important factor that could influence macrophage polarization and responses is macrophage metabolism and the metabolic flux through different metabolic pathways (i.e., glycolysis, glucose oxidation, fatty acid oxidation, ketone oxidation, and amino acid oxidation) [9]. However, we still do not know the metabolic profiles for the different polarization states of macrophages. Several studies have also linked macrophage activity with the development of various cardiometabolic diseases, including heart failure, obesity, and diabetes [10–12]. In addition, macrophage activation is associated with sustained inflammatory responses characterized by the secretion of chemokines, cytokines, and matrix protein-degrading enzymes that could exacerbate cardiometabolic diseases via exacerbating inflammation [12–14]. However, how these cardiometabolic diseases influence macrophage energy metabolism and activity is still unclear. Here, we conducted a comprehensive review of the literature to summarize the current understanding of the alterations in macrophage metabolism in cardiometabolic diseases and how that might contribute to the development and progress of these diseases.

## Macrophage polarization

The activity of a macrophage depends on its responsiveness to the surrounding tissue microenvironment and the immuno-metabolic alterations that are triggered as a result. This phenomenon, known as macrophage polarization, encompasses the ability of macrophages to adopt specific functional states with specialized roles in immune response and tissue homeostasis in response to environmental cues and homeostatic conditions. Earlier studies have shown that macrophages polarize into two phenotypes: classically activated/pro-inflammatory/M1 and alternatively activated anti-inflammatory M2 [15]. This classification was introduced by Charlie Mills in 2000 [15], inspired by the Th1/Th2 paradigm by Mosmann and Coffman in 1986 [16]. Several key signaling pathways and regulatory networks modulate the programming process of polarization into each type. Cell surface marker expression is also a key indicator of macrophage polarization [17]. For example, M1 macrophages tend to greatly express CD16, CD38, CD68, CD80, and CD86 [18]. Contrarily, M2 macrophages show elevated arginase-1, CD206, CD163 and IL-10 expressions [19]. M1/M2 polarization is also based on the observation that lipopolysaccharides (LPS) and interferon-γ (INF-γ) elicit different responses in macrophages harvested from different mouse strains [15]. The study showed that macrophages isolated from the so-called Th1 strains (C57BL/6) produce nitric oxide (NO) in large quantities, while the same triggers stimulate arginine metabolism to ornithine in macrophages isolated from Th2 strains (Balb/c). The ability of macrophages to adopt specific confirmations and functions in response to various cues is crucial for modulating inflammatory responses and healing processes. However, the binary classification of M1 and M2 macrophages has been challenged by recent studies demonstrating that macrophages’ polarization and metabolic profiles are in a continuous spectrum in response to various stimuli [20]. Therefore, we will confine the nomenclature to M1-like and M2-like macrophages. M1- and M2-like macrophages carry out different responsibilities, where M1-like macrophages are mostly involved in the first line of defense, whereas M2-like macrophages are mostly involved in the longer-term resolution. The distinct metabolic profiles between M1- and M2-like macrophages are built to support their different actions [20].

It should also be noted that although the concept of M1/M2 macrophage polarization was initially characterized in mice, significant species–species differences may also exist. Both human and mouse macrophages exhibit M1-like and M2-like phenotypes, but the markers, signaling pathways, and polarization stimuli can differ [21, 22]. For example, distinct differences at the transcriptome level indicate that arginase-1 (Arg-1) and chitinase-like protein 3 (Ym1) are markers for murine but not for humans in alternatively activated myeloid cells [23]. In addition, a known major difference between mouse and human macrophages is arginine metabolism [24]. Mouse macrophages metabolize arginine predominantly by inducible nitric oxide synthase (iNOS) and Arg-1, which can promote either M1 or M2 polarization [25]. In contrast, human macrophages exhibit similar pathways, but Arg-1 expression is less prominent and cannot be induced by LPS or IL-4, which affects the overall balance of arginine metabolism and resulting macrophage function [26, 27]. Using quantitative proteome analysis, a recent study by Li et al. demonstrated significant divergences of polarized macrophages from human THP-1 and mouse RAW264.7 cell lines [28]. Human THP-1 cell-derived M2-like macrophages rely on oxidative glucose metabolism and fatty acid oxidation [28], contradicting earlier evidence that M2-like macrophages only rely on fatty acid oxidation to support their polarization [29]. Taken together, these findings underscore the gap in knowledge regarding the different metabolic profiles of M1/M2 subtypes and the main differences between humans and murine macrophages. Although the M1-like and M2-like paradigm provides a useful framework, it is essential to consider interspecies differences, the broader spectrum of macrophage activation states, and how the metabolic profile of M1/M2 subtypes might differ.

### Activation of M1-like macrophages

M1-like polarization of macrophages can be induced by pro-inflammatory cytokines and microbial products, such as bacterial LPS, which may stimulate TLRs [30]. TLRs are transmembrane PRRs expressed on the surface of macrophages for ligand recognition [31]. When TLRs are activated, the macrophage undergoes metabolic reprogramming caused by intracellular signaling cascades [31]. For example, the activation of TLR4 led to a quick increase in glucose uptake, glycolysis, tricarboxylic acid (TCA) cycle activity, and citrate generation [32]. Through metabolic tracing, studies have shown that LPS signaling promotes the incorporation of acetyl-Coenzyme A (CoA) into histones, which leads to increased histone acetylation [32].

M1-like macrophages produce pro-inflammatory cytokines and interleukins, such as IL-1 β, IL-6, and tumor necrosis factor-alpha (TNF-α), with an increase in iNOS expression [33]. As a part of the innate immune response associated with macrophages, chemokines, interferons, and ROS are also released [33]. A by-product produced in M1-like macrophages is NO, which induces oxidative stress at high concentrations and, overall, leads to damage to DNA and the inhibition of DNA synthesis as a component of microbicidal activity [34]. IFN- γ is a crucial Th1-cells-derived inflammatory mediator that influences the polarization of the M1 phenotype and is associated with the overproduction of pro-inflammatory cytokines, including TNF-α and IL-6. When IFN- γ binds to IFN- γ receptors (IFN- γR), the Janus kinases are activated, which causes the activation of STAT1 [35]. The overall polarization of the M1 phenotype causes a change in the metabolic profile in which the anaerobic glycolytic pathway is upregulated to provide the necessary energy and metabolic intermediates necessary for the synthesis of pro-inflammatory molecules in the polarized macrophage [36, 37]. This is evidenced by increased hexokinase activity and elevated glucose-6-phosphate dehydrogenase (G6PD) expression, which supports an overall increase in glycolysis and the pentose phosphate pathway [38].

Hypoxia-inducible factor (HIF-1α) is a central regulator in immune responses and a participant in the induction of M1 polarization [39–41]. Studies have shown that the overexpression of HIF-1α led to increased metabolic intermediates from glycolysis and the pentose phosphate pathway [39]. HIF-1α-induced differentiation to M1-like macrophage is shown to be caused by the upregulation of mRNA expression of pyruvate dehydrogenase kinase 1 (Pdk1), phosphoglycerate kinase 1 (Pgk1), glucose transporter 1 (Glut1), glucokinase (Gck), and pyruvate kinase M2 (Pkm2) [39]. The resulting increased glycolysis allows for sufficient energy production and the creation of metabolic intermediates necessary to meet the energy and biosynthetic demands for the secretion of pro-inflammatory cytokines in pro-inflammatory macrophage polarization [39].

Another key pathway in macrophage polarization is the mechanistic target of rapamycin (mTOR), which consists of 2 main complexes, mTORC1 and mTORC2 [42]. The mTORC1 signaling cascade plays an important role in regulating energy metabolism, adipogenesis, protein synthesis, and lysosomal formation processes, while the mTORC2 signaling cascade primarily influences cell survival and metabolism [42]. The polarization of M1 macrophages, along with metabolic reprogramming, can be regulated by mTORC1 through activating gene transcription of several factors and proteins and playing a role in the transcription of pro-inflammatory cytokines and glycolytic genes as part of the mTORC1/HIF-1α axis [43]. Wu et al. demonstrated that pharmacological inhibition of the mTORC1/ HIF-1α axis reduces glycolytic rates and M1-like polarization in macrophages [44]. The presence of the tuberous sclerosis complex (TSC) is also a key regulator of mTOR-related macrophage polarization through inhibiting mTORC1 signaling and thus causing inhibition of M1-like polarization through inhibiting Ras GTPase–Raf1–MEK1/2–extracellular signal-regulated kinase (ERK) signaling [45]. In TSC1-deficient macrophages, Ras GTPase activity is enhanced and thus causes high MEK-ERK activation, which promotes M1-like polarization [45]. TSC1 deletion in bone marrow-derived macrophages (BMDMs) enhances M1-like activation in the presence of LPS [45, 46]. This was supported by enhanced inflammatory response, increased pro-inflammatory cytokine secretion, increased NO production, and downregulation of Arg-1 and macrophage galactose-type lectin-1 (Mgl1) in the M2-like phenotype [45, 46]. However, it is still unclear whether downregulated mTORC2 signaling also contributes to the production of cytokines in TSC1-deficient macrophages. Of relevance is that TSC1 promotes M2-like polarization through the mTOR-dependent CCAAT/enhancer-binding protein-β pathway [45]. It has been shown that TSC1 knockout macrophages have reduced M2-like activation with decreased expression of M2 markers, including Arg-1, Ym1, and Fizz (found in the inflammatory zone)1 [45]. Myeloid-specific TSC1 deletion in macrophages decreases fatty acid oxidation and Arg-1 activity, essential components of the M2-like metabolic program [47]. Further investigation into the influence of TSC in regulating macrophage polarization through mTOR-related pathways would be a point of interest in developing therapeutic strategies.

### Activation of M2-like macrophages

In damaged tissue, macrophages exhibit wound healing and tissue regeneration functions formed by M2-like macrophages [26]. The M2 state is akin to the job of a repairman, where the aim is to repair tissue and remove debris to induce wound healing. The main stimulating factors in the M2-like polarization of macrophages are Th2-derived cytokines, including IL-4/IL-13, which activates signal transducer and activator of transcription 6 (STAT6) through binding to the IL- 4Rα receptor [26]. IL-10 also activates the signal transducer and activator of transcription 3 (STAT3) through the IL-10 receptor to induce M2 polarization [48, 49]. As a consequence of M2 differentiation, there is an overall metabolic shift toward greater employment of fatty acid oxidation and oxidative phosphorylation (OXPHOS) for the production of anti-inflammatory factors, including IL-10, TGF-β, and IL-1Ra, to dampen inflammatory immune responses and promote tissue repair [50].

IL-4 is an important trigger of fatty acid uptake and plays a role in upregulating genes relevant to oxidative metabolism in macrophages. The sustenance of the IL-4-induced M2-like metabolic program is mediated through feed-forward control of STAT6 and peroxisome proliferator-activated receptor-gamma coactivator 1β (PGC-1β) [51]. Stimulating macrophages with IL-4 activates the fatty acid metabolism program by upregulating oxidative genes such as medium-chain acyl-CoA dehydrogenase (MCAD) [51]. Through the usage of mitochondrial inhibitors (etomoxir, oligomycin) in BMDMs, the lack of mitochondrial oxidative metabolism significantly reduced the anti-inflammatory effects of IL-4 and uncoupling of mitochondrial respiration prevented IL-4-induced arginase activity [51]. However, whether fatty acid oxidation is required for M2-like polarization is still debated. It has been suggested that fatty acid oxidation is critical for M2-like differentiation in studies utilizing pharmacological inhibition of carnitine palmitoyltransferase I (CPT1) with etomoxir [52, 53] or CPT1a knockdown [54]. Conversely, other studies have shown that inhibiting macrophage fatty acid oxidation in carnitine palmitoyltransferase II (CPT2) knockout mice does not affect M2-like polarization following IL-4 stimulation in vitro and in vivo [55, 56]. In line with that, IL-4 exposure is shown to trigger macrophage polarization with M2-like macrophage markers (CD206, CD71, CD301) and M2-associated gene expression (Mgl2, Relmα, Ym1, Fabp4, Arg-1) in the presence of etomoxir [57]. Similar results are also reported in human macrophages [22], suggesting that the role of fatty acid oxidation in M2-like polarization may be more complex than initially thought and highlighting the need for further investigation into whether fatty acid oxidation is correlative or causal in macrophage polarization.

Within the M2-like categorization, there are four additional subgroups. T-helper 2 cytokines such as IL-4 or IL-13 polarize macrophages into the M2-like phenotype and promote tissue repair, cell growth, and endocytic activity, creating the M2a subtype [58, 59]. If immune complexes such as human myeloma immunoglobulin G1 (mlgG1), toll-like receptor ligands, or IL-1 β are introduced to M2a macrophages, the focus of the macrophage would be to instead regulate immune responses within the environment through pro- and anti-inflammatory cytokine production, leading to the M2b subtype [60, 61]. M2c macrophages, also known as inactivated macrophages, are created by glucocorticoid and IL-10 exposure, and they play a major role in apoptotic cell phagocytosis [62]. The final subtype is M2d macrophages, which are known as tumor-associated macrophages (TAMs) and are induced by Toll-like receptor (TLR) agonists through the activation of adenosine A2A receptors [63, 64]. The main contribution of M2d is the promotion of angiogenesis and heightened secretion of IL-10 and vascular endothelial growth factors (VEGF) [64, 65].

The insulin signaling pathway also plays a role in triggering the activity of M2-like macrophages. For instance, activation of the phosphatidylinositol-3-kinase (PI3K)—protein kinase B (Akt) signaling pathway can inhibit pro-inflammatory while promoting anti-inflammatory responses in toll-like receptor (TLR)-stimulated macrophages [66]. The overexpression of PI3K or Akt kinases decreases LPS activation of macrophages [67, 68]. However, it has also been shown that M1-like polarization can occur through PI3K/Akt activation. Arranz et al. have shown in vitro and in vivo that inhibiting the Akt1 isoform induces M1-like polarization while inhibiting the Akt2 isoform induces M2-like polarization [69]. As the polarization of macrophages through the PI3K/Akt pathway is isoform dependent, modulation of macrophage phenotype may be possible through inhibiting certain Akt kinase isoforms. Activating this pathway also plays an indispensable role in glycolytic usage in macrophages. Chang et al. have shown that oil-in-water emulsion-stimulated glucose uptake relies on PI3K activity in BMDMs due to the upregulation of Glut1 levels [70]. It has also been shown that pharmacological inhibition of PI3K reduces glucose uptake by ~ 45% in colony-stimulating factor 1 (CSF-1)-deprived BMDMs, emphasizing the importance of PI3K activity in glucose uptake [70]. In further support of this, Huang et al. have also demonstrated that PI3K, Akt, and mTORC2 are crucial in the metabolic changes needed for macrophage M2-like activation, especially their roles in enhancing glucose metabolism in macrophages [54].

A key biomarker of M2-like activation is increased levels of arginase-1, which breaks down arginine to urea and ornithine, which reduces NO production and helps sustain regenerative processes [71, 72]. In the M1-like phenotype, iNOS is upregulated with LPS or IFN-γ stimulation and metabolizes arginine to produce NO, but arginine can also be metabolized by arginase into ornithine and urea, which can be used in the urea cycle [73, 74]. The relative expression of iNOS to Arg-1 is pivotal in mediating arginine catabolism, contributing to the distinct functions of macrophage polarization. The nuclear receptor, peroxisome proliferator receptor-gamma (PPARγ), is important in enhancing macrophage polarization to the M2-like phenotype and inhibiting the M1-like phenotype [75]. For instance, Odegaard et al. have shown defective polarization of M2-like macrophages in macrophage-specific PPARγ-deficient mice [75]. The lack of PPARγ in macrophages causes a significant reduction in arginase-1 activity, the expression of mitochondrial biogenesis transcription factors (Tfam, Nrf-1), and the expression of mRNAs encoding enzymes in fatty acid oxidation (Cpt1b, Acox1) and OXPHOS (Ndufs1, Sdh, Atp5j, Atp5b) [75]. These findings highlight the critical role of PPARγ in regulating macrophage polarization and function and that targeting PPARγ could be a potential therapeutic strategy to control macrophage polarization [75].

It has been reported that the upregulation of glutamine synthetase (GS) plays a role in modulating the M2-like polarization of macrophages. For example, IL-10-induced expression of M2 markers and promoted increased pro-inflammatory mediators such as iNOS are blocked in macrophage-specific GS knockout mice [76]. GS deletion was also associated with reduced intracellular glutamine, increased succinate accumulation, enhanced glucose flux, and increased HIF-1α activity, a key participant in M1-like activation [76]. In another study, glutamine deprivation negatively affects M2-like polarization in mouse BMDMs [77]. Depleting glutamine in macrophages reduces M2-like polarization, evidenced by decreased expression of M2-like activation markers (CD206, CD301, Relmα) [77]. Polarized M2-like macrophages in glutamine-deprived media also expressed downregulated M2-specific marker genes such as Irf4 and Ccl22 and downregulated TCA cycle activity through gene set enrichment analysis [77]. Taken together, these studies demonstrate the relevancy of glutamate to glutamine conversion and glutamine metabolism in promoting the immunosuppressive M2-like state.

## Immuno-metabolic control of macrophage activation

As macrophages are responsible for combating bacterial infection and immune development processes, they need to generate enough energy to support their cellular growth, survival, and proliferation [78]. Macrophages use several metabolic pathways for energy production, including glycolysis, fatty acid oxidation, glucose oxidation, amino acid oxidation, ketone oxidation, and the pentose phosphate pathway [1, 79]. These metabolic pathways can all contribute to adenosine triphosphate (ATP) production in different proportions depending on the energy requirement of the macrophages and their differentiation status [20]. In addition, the various metabolites generated from different metabolic pathways are also involved in regulating inflammatory responses to synchronize with the cellular needs of the macrophages [78].

### Metabolic regulation of M1-like macrophage

Earlier studies have suggested that M1-like macrophages are characterized as gearing toward anaerobic glycolysis with attenuated activities of the electron transport chain (ETC) [20]. This was justified by the physiological relevance of M1-like macrophages acting as the first line of defense to combat micro-organism infection. This process needs a quick turn-out of ATP production, and this is usually supported with glycolysis, an anaerobic process that can occur rapidly to generate 2 ATP molecules per glucose consumed [80].

Glycolysis is the first step of glucose metabolism. This metabolic pathway does not require oxygen to generate ATP and results in limited ATP production (i.e., only 2 ATP molecules per unit of glucose). The anaerobic nature of glycolysis is crucial for macrophages as they are usually found in sites with low oxygen levels, such as in tumors and sites of infection. The metabolites produced along the glycolytic pathway are also shown to be essential for biosynthetic growth and proliferation. For example, the activation of the glycolytic pathway is supported by the switch of expression of the liver form 6- se-2-kinase/fructose-2,6-bisphosphatase (L-PFK2) to the ubiquitous form (u-PFK2) [5]. PFK2 converts fructose-6-phosphoate to fructose-2,6-biophosphate, which activates phosphofructokinase-1 (PFK1). This enhances the conversion of fructose-1.6-biphosphate to pyruvate as the end product of the glycolytic pathway. Glucose entering the glycolytic pathway could be rerouted to the parallel pentose phosphate pathway, leading to the generation of NADPH. Oxidation of NADPH is associated with ROS production, which is essential for supporting the action of M1-like macrophages on phagocytosis [34]. In addition, a few glycolytic enzymes are also involved in supporting the pro-inflammatory M1-like macrophage functions. For example, pyruvate kinase M2 (PKM2) can activate HIF-1α to stimulate IL-1β expression while also stimulating eukaryotic translation initiation factor 2 alpha kinase 2 (EIF2AK2) for inflammasome activation [81, 82]. Using macrophages isolated from patients with atherosclerotic coronary artery disease, it has been demonstrated that increase in dimerization of PKM2 facilitates its nuclear translocation. Nuclear PKM2 can phosphorylate transcription factors like STAT3, leading to increased production of pro-inflammatory cytokines, such as IL-6 and IL-1β [83].

The capacity of OXPHOS and TCA cycle is shown to be altered in M1-like macrophages [20]. Specifically, the TCA cycle is interrupted at two points. The first break occurs at isocitrate dehydrogenase, which converts isocitrate to α-ketoglutarate (α-KG). This leads to an accumulation of isocitrate and its precursor citrate within the mitochondria. This build-up of citrate leads to a secondary blocked point along the TCA cycle at the step of succinate dehydrogenase [84], which converts succinate to fumarate. This is because citrate can be converted to itaconate, a competitive inhibitor for succinate dehydrogenase. Blockage at succinate dehydrogenase could also lead to the accumulation of succinate. It has been reported that succinate accumulation is critical in activating HIF1α in response to LPS induction of M1-like polarization [85]. Activation of HIF-1α can drive the production of pro-inflammatory cytokines, such as IL-1β [85]. Additionally, succinate dehydrogenase is an important unit for complex II of the ETC, which transfers protons to build up membrane potential to drive the action of ATP synthase [86]. As such, the overall mitochondrial respiratory capacity is limited in M1-like macrophages due to the blockage at complex II [84, 87, 88]. Succinate dehydrogenase activity has also been implicated in increased production of ROS as electrons can reversely flow back to complex I [89].

Amino acids are another type of energy substrate that macrophages can metabolize. For example, arginine metabolism is important in determining whether macrophages polarize to M1-like or M2-like phenotype [74]. In M1-like macrophages, arginine is mostly metabolized to produce NO, as supported by the upregulated activity of iNOS [74]. iNOS is transcriptionally regulated by HIF1α, which is activated in M1-like macrophages [90]. The produced NO could, in turn, further suppress OXPHOS by inhibiting complex I and IV activities [91, 92]. In M2-like macrophages, arginine is processed differently by arginase-1, which converts arginine into ornithine. Ornithine can then be further converted into proline and polyamines [93].

Earlier studies proposed that M1-like macrophages solely use glycolysis to support their functions by suppressing fatty acid oxidation and OXPHOS [94]. However, recent studies have challenged these suggestions, showing that fatty acid oxidation also occurs in M1-like macrophages [94]. M1-like macrophages are more geared toward fatty acid synthesis, producing substances like prostaglandins and leukotrienes to serve as inflammatory mediators [94]. Fatty acid synthesis is crucial for maintaining the pro-inflammatory phenotypes of M1-like macrophage inflammatory gene expressions, and recruitment of macrophage to adipose tissue in response to high-fat diet feeding is attenuated in myeloid cell-specific fatty acid synthase (FAS) knockdown mice [95]. In addition, FAS deficiency correlates with ameliorated insulin resistance and diminished chronic inflammation, suggesting the potential of targeting fatty acid synthesis in M1-like macrophages to alter its polarization status and associated physiological effects [95].

### Metabolic regulation of M2-like macrophage

M2-like macrophages are functionally more long-lasting and play roles in resolution and repairment [52]. Earlier studies have suggested that increased reliance on fatty acid oxidation is a main characteristic feature of M2-like macrophages [94]. Fatty acid oxidation has the potential to yield over 100 ATP per unit of fatty acid molecule. Fatty acid is firstly converted to fatty acid acyl-CoA in the cytosol, followed by conjugation with the carnitine moiety via carnitine palmitoyl transferase I (CPT1), which enables fatty acid to bypass the mitochondrial membrane. Once inside the mitochondria, carnitine moiety is removed from fatty acylcarnitine to regenerate acyl-CoA, which undergoes β-oxidation. Vats et al. also showed that knockdown of PGC1β partially inhibits IL-4-induced upregulation of fatty acid oxidation [51]. More importantly, pharmacological inhibition of fatty acid oxidation with etomoxir completely abolishes M2 polarization [55]. Interestingly, α-KG is a positive regulator of fatty acid oxidation [96], suggesting the crosstalk among different metabolic pathways that collectively assist M2 polarization.

While glycolysis is shown to be upregulated in M1-like macrophages, studies have shown that M2-like macrophages could also rely on glycolysis for energy production and polarization. Some studies have shown that macrophages’ polarization to an M2-like state is impaired when using 2-deoxyglucose (2-DG), a glucose-mimicking molecule that can be uptake into the cell but cannot undergo further glycolysis due to the replacement of hydrogen with a hydroxyl group [97]. On the contrary, other studies proposed that 2-DG inhibits glycolysis and diminishes OXPHOS and intracellular ATP levels [98]. Of relevance is that glucose substitution with galactose effectively suppresses glycolytic activity but has no effective suppression of OXPHOS or M2 differentiation marker expression [99]. Therefore, these findings suggest that glycolysis is unnecessary but represents an extra source of ATP for M2-like macrophages. Evidently, M2-like macrophages can tightly regulate the rate of glycolysis by modulating the expression of the glycolytic enzyme 6-phosphofructo-2-kinase B1 (PFKFB1), which converts fructose-2,6-bisphosphate to fructose-6-phosphate [79]. Fructose-2,6-bisphosphate is an endogenous activator of glycolysis; as such, the action of PFKFB1 in catabolizing fructose-2,6-bisphosphate can facilitate the control of glycolytic rates. An intact TCA cycle is crucial for M2-like macrophages to sustain energy production and biosynthetic processes [50]. The TCA cycle uses acetyl CoA, which can be generated from the oxidation of glucose, fatty acid, amino acids, and ketones, for producing reduced equivalents, NADH and FADH2, which will feed into the ETC for ATP production. Additionally, intermediates of the TCA cycle, such as oxaloacetate, are essential precursors for the synthesis of nucleotides, the building block for cellular DNA and RNA, which will be relevant for M2-like macrophages as their primary role is involved in tissue repair and resolution of inflammation [50].

The differential use of glutamine can determine the polarization of macrophages into either M1- or M2-like phenotypes. In M1-like macrophages, glutamine is mostly channeled into the TCA cycle to synthesize succinate, which can stabilize HIF-1α to promote polarization to M1 phenotypes [100]. On the other hand, in M2-like macrophages, glutamine is converted to α-ketoglutarate through glutaminolysis. α-KG can promote M2 polarization by providing a substrate for the TCA cycle and UDP-GlcNAc synthesis [100]. UDP-GlcNAc is an important molecule used for N-glycosylation, which can modify protein structure and function that are abundantly expressed in M2-like macrophages [77]. Using radiolabeled isotopes tracing technique (^15^N-glutamine), it has been shown that glutamine is attributed for more than half of the nitrogen in UDP-GlcNAc [77]. As glutamine metabolism is paramount for the M2 phenotype, macrophages are equipped to self-generate glutamine from precursor glutamate and ammonia via glutamine synthetase (GS). GS is found to be highly expressed in M2-like macrophages but is minimally detected in M1-like macrophages [76]. The role of GS in synthesizing glutamine is essential for the acquisition of M2 phenotypes, as supported by the observation that inhibition of GS reprograms M2-like macrophages to M1-like phenotypes [50].

Although it still remains unclear, ketone metabolism has been shown to be relevant for polarizing M2-like macrophages [36, 101–103]. Ketones are organic compounds with a chemical structure characterized by a carbonyl group bonded to two carbon atoms. The liver can produce ketones by breaking down fatty acid molecules, primarily during fasting or when circulating glucose levels drop. β-hydroxybutyrate (βOHB), acetoacetate (AcAc), and acetone are the three major forms of ketones. Notably, βOHB has been shown to promote M2 polarization and provides beneficial effects in the context of inflammatory bowel disease [101]. In a mouse model of acute dextran sulfate sodium-induced colitis, administration of βOHB resulted in increased expression of IL-10 and Arg-1, both are canonical markers of M2 polarization, which is accompanied by resolution of intestinal inflammation and the repair of damaged intestinal tissues [101]. Using isotope tracking LC/MS untargeted metabolomics, a recent study has shown that both M1- and M2-like macrophages selectively oxidize acetoacetate but not βOHB [104]. Furthermore, exogenous AcAc supplements have been shown to ameliorate diet-induced hepatic fibrosis in a macrophage-dependent manner [104].

## Alterations in macrophage metabolism in cardiometabolic diseases

### Obesity

Obesity is a major health concern, as an estimated ~ 641 million adults worldwide are obese [9, 105, 106]. A major factor contributing to this condition is the development of insulin resistance, which involves the impairment of the insulin signaling pathway and has been linked to metabolic inflammation [10, 107]. Studies have shown that macrophages can increase from 10 to 50% of total cells in obese subjects [108]. This is likely due to increased monocyte-derived macrophage infiltration into the heart [109]. However, how macrophage energy metabolism is altered in obesity and how that influences their infiltration into the myocardium in obesity is understudied and still not fully understood.

#### Glucose

In obesity, whole-body insulin resistance is greatly affected by the activity of adipose tissue macrophages (ATM), the main producers of inflammatory cytokines in adipose tissue [110]. In obese mice, specific depletion of phagocytic cells by intraperitoneal injection of clodronate liposomes enhanced insulin sensitivity and glucose homeostasis, decreased plasma TNFα, and increased plasma adiponectin [111, 112]. These studies highlight the contribution of ATM to the whole-body insulin resistance and inflammation in obesity. Yet, it is not clear what phenotype ATM develops in obesity. Insulin signaling influences macrophage action by controlling glucose uptake and metabolism [113]. Macrophages have functional insulin signaling, and they develop insulin resistance in the context of systemic insulin resistance [113]. Ieronymaki et al. have shown that insulin-resistant macrophages display attenuated the Akt2/mTORC1 signaling pathway in diet-induced glucose-intolerant mice [113]. Despite this reduction in insulin signaling, glycolysis is notably increased in insulin-resistant macrophages [113].

A metabolic product of glucose metabolism, lactate, and expression of glycolytic enzymes (hexokinase 3, kinase, lactate dehydrogenase) are also significantly elevated in insulin-resistant macrophages [113]. In line with that, the expression of Glut1 and Glut3, the primary glucose transporters expressed in macrophages which respond to insulin receptor and insulin-like growth factor 1 receptor signals, are also altered in insulin-resistant macrophages [113]. Other studies have suggested that the unique rewiring of intracellular energy metabolism of ATM consists of upregulation of both glycolysis and OXPHOS, distinct from both M1-like and M2-like phenotypes. Boutens et al. demonstrated that there is an upregulation of genes involved in OXPHOS (Atp6v0d2, TCIRG1, Atp6v1b2) and glycolysis (ENO2, HK3, HK2, PKM) in ATMs [10]. In addition, BMDMs exhibit enhanced glycolysis and OXPHOS when co-cultured with obese adipose tissue, highlighting the importance of the adipose tissue environment in driving metabolic changes [10]. Pharmacological inhibition of glycolysis in ATMs with 2-DG reduces lactate levels and limits cytokine release in obese mice [10]. This demonstrates how cytokine release by ATM in obesity is influenced by glycolysis. Similar metabolic signatures of elevated glycolysis and OXPHOS linked to pro-inflammatory markers have also been seen in human macrophages isolated from adipose tissue of obese individuals with type 2 diabetes [10].

The metabolic end-product of glycolysis, l-lactate, is also a critical molecule that could influence macrophage metabolism in obesity [114]. Long-term l-lactate administration improves insulin resistance and suppresses M1-like polarization by activating the GPR132-PKA-AMPK α1 signal in macrophages in high-fat diet-induced obesity [114]. Contrarily, it has been shown that single doses of l-lactate induce insulin resistance in skeletal muscle [115]. Lin et al. have also demonstrated that lactate accumulation triggers the obesity-induced inflammatory response in adipose tissues [116]. Slc16a1 is a gene that encodes MCT1, a key lactate transporter in adipose tissue. In a mouse model of high-fat diet-induced obesity, deletion of Slc16a1 causes lactate accumulation and aggravates systemic insulin resistance, macrophage recruitment, and cytokine expression [116]. Therefore, data regarding the role of l-lactate in modulating macrophage responses and insulin sensitivity in obesity are inconclusive.

The activity of HIF-1α is increased in adipose tissue in obesity [117]. HIF-1α is known for promoting a glycolytic pro-inflammatory phenotype in macrophages [39]. This increased expression of HIF-1α leads to greater recruitment of M1-like macrophages that secrete pro-inflammatory cytokines, connective tissue growth factor, NADPH oxidase, pro α2 (I) collagen, and growth factor β [117]. HIF-1α also assists in macrophages’ activation, migration, and adhesion into the epicardial adipose tissue [117]. These secretions affect extracellular matrix synthesis, contribute to the aggravation of myocardium fibrosis, and impair cardiac function [117]. The inhibition of HIF-1 α in obese mice reduced inflammation in the adipose tissue and impaired macrophage recruitment [117]. Therefore, targeting HIF-1α and the associated pro-inflammatory macrophage response represent a potential therapeutic approach to limit the detrimental effect of obesity on the heart.

#### Fatty acids

Lipid metabolism is also altered in ATMs with elevated rates of lysosomal biogenesis and lysosomal-dependent lipid catabolism, induced by adipose tissue factors and linked to lipid accumulation [118]. The expressions of lysosome-associated membrane protein 2 (LAMP2) and lysosomal acid lipase A (LIPA) are increased in obese mice and obese human subjects [118]. In obesity, local lipid concentrations are increased due to hypertrophy and apoptosis of adipocytes, which leads to the accumulation of ATMs [118]. To buffer the lipotoxic effects, extracellular lipids are taken in by ATMs to undergo lipid catabolism in lysosomes. This is essential for lipophagy and exophagy, two important lysosome-dependent pathways for regulating cholesterol efflux and degradation of adipocyte debris. Lysosome function is also shown to be necessary for lipid catabolism in ATMs as inhibition of lysosome function by chloroquine and bafilomycin A1 increases lipid droplet formation and reduces lysosomes [118].

Inflammation in adipose tissue is marked by M1-like macrophage activation, which triggers ROS generation and pro-inflammatory cytokine secretion [119]. Chronic inflammation associated with obesity often plays a major role in the pathogenesis of cardiomyopathy. Obesity is associated with increased accumulation of macrophages in the epicardial adipose tissue (EAT) [120]. EAT is a major source of anti-inflammatory and pro-inflammatory adipokines, which greatly impact cardiac function [121]. It has been found that the ratio of M1-like and M2-like macrophages in human EAT is linked to the development of coronary artery disease [121]. In the epicardial fat of patients with CAD, the CD11C/CD206 ratios and the levels of inflammatory cytokines, including IL-6, TNF-α, and MCP-1, are increased, indicating an increase in M1-like macrophages and a shift toward an inflammatory response [121]. Saturated fatty acids, released in large quantities from hypertrophied adipocytes via macrophage-induced adipocyte lipolysis, contribute to the obesity-induced inflammatory state in the vascular wall by triggering the TLR4/NF-κB pathway [122–124]. Through an in vitro co-culture system of adipocytes and macrophages, Suganami et al. demonstrated that pharmacological suppression of the NF-κB pathway leads to inhibition of co-culture-induced lipolysis in adipocytes [122]. These findings show how the NF-κB pathway regulates fatty acid release in obese adipose tissue [122]. Coculturing hypertrophied adipocytes with TLR4-deficient macrophages also leads to the inhibition of pro-inflammatory markers and lipolysis [122]. The interaction of adipocytes and macrophages in the TLR4/NF-κB pathway may be a point of interest in alleviating obesity-induced inflammation in the heart and other organs.

The depressed mitochondrial function causes a metabolic shift in macrophage fatty acid metabolism toward increased triglyceride, phospholipid, and ceramide synthesis, collectively increasing lipotoxicity in macrophages [125, 126]. Consequently, mitochondrial dysfunction in ATMs is also associated with NLRP3 inflammasome activation and IL-1β release [126, 127]. IL-1β plays a key role in promoting the activation of the IκB kinase complex (IKK)/ nuclear factor (NF)-κB pathway, which leads to insulin resistance through IKK-mediated serine phosphorylation of insulin receptor substrate-1 (IRS-1), thus causing inhibition of tyrosine phosphorylation of IRS-1 by the insulin receptor and impairment of downstream signaling [128, 129]. In addition, IL-1β also activates c-Jun NH(2)-terminal kinases and other mitogen-activated protein kinases (MAPKs) to further induce insulin resistance by impairing the interaction between IRS and downstream insulin signaling [130, 131].

Sterol regulatory element-binding protein cleavage-activating protein (SCAP) is an escort protein that participates in sterol regulatory element-binding protein (SREBP) movement regulation, which controls cholesterol synthesis and lipid metabolism in macrophages [132]. In macrophage-specific SCAP knockout obese mice, it has been shown that fat accumulation is elevated in both liver macrophages and ATMs [132]. Proinflammatory macrophage infiltration in adipose tissue induced by high-fat and high sucrose diet is also increased by SCAP deletion, highlighting the role SCAP in determining macrophage phenotype and associated cytokines secretion in obesity and other metabolic diseases [132]. The role of SCAP activity in macrophages and how it influences macrophage metabolism and polarization in cardiometabolic diseases warrants further investigation. The protein-triggering receptor expressed on myeloid cells 2 (Trem2) is shown to play a large role in tissue-level immune cell remodeling by driving lipid catabolism and energy metabolism [133]. A subset of ATMs, namely CD9^+^CD63^+^ macrophages, are known as lipid-associated macrophages. These macrophages are characterized by Trem2 expression, which plays a role in sensing extracellular lipids and their protective effects by counteracting metabolic dysfunction [133]. Genetic deletion of Trem2 in obese mice worsens body fat accumulation, glucose intolerance, hypercholesterolemia, and increased cholesterol levels [133]. This suggests that macrophages expressing Trem2 may be important in maintaining the metabolic health of the whole body.

#### Ketones

Ketone bodies are compounds primarily synthesized in the liver through the breakdown of fatty acids. While regulation of ketone metabolism in macrophages and its alteration in obesity remains poorly understood, recent isotope tracking LC/MS untargeted metabolomics revealed that macrophages preferentially oxidize AcAc over βOHB [104]. Clinical studies have also demonstrated that the improvement in diet-induced hepatic fibrosis due to increased circulating AcAc levels is reversed in macrophage-specific SCOT KO mice [104]. These findings highlight the critical need for further investigation into the role of macrophage ketone oxidation in modulating inflammatory responses.

#### Amino acids

Glutamine, crucial for cellular carbon and nitrogen, shows altered metabolism in macrophages exposed to M1/M2-like polarizing agents [134]. In obese insulin-resistant conditions, macrophages collected from Zucker rats exhibit reduced NO production, but supplementation with glutamine enhances NO synthesis [135]. This suggests the existence of an interplay between arginine and glutamine metabolism in modulating macrophage responses, as NO is produced using arginine as a precursor. However, in macrophages, the specific role of amino acid metabolism and the differential effects of various amino acids in obesity-induced inflammation have yet to be fully understood.

### Type 2 diabetes

Type 2 diabetes (T2D) continues to be a growing health concern worldwide as the number of people diagnosed with T2D has increased at a steady rate over the past few decades and is predicted to reach around 642 million globally by the year 2040 [136]. T2D is characterized as a metabolic disorder with low-grade inflammation [137]. Inflammation is known to trigger monocyte differentiation into macrophages. Progress of complications associated with T2D is also dependent on the build-up of macrophages in tissues vulnerable to diabetic injury. However, it is not clear how alterations in macrophage energy metabolism might contribute to monocyte- and macrophage-mediated injury recruitment in complications associated with T2D.

#### Glucose

Macrophages are sensitive to insulin and changes in oxidative substrate availability [4]. This is relevant for T2D, as plasma glucose levels are elevated in T2D due to poorly controlled glucose clearance. While it is still unclear whether these high circulating glucose levels enhance glucose uptake in macrophages, glycolytic rates are upregulated in adipose tissue macrophages of obese mice and rats [10]. This increase in glycolysis is supported by an increase in Glut1 expression, which facilitates more glucose uptake into macrophages. Similarly, ATMs of obese patients also have higher lactate levels, which indirectly suggests higher glycolytic rates and/or impaired mitochondrial glucose oxidation compared to adipose tissue macrophages from lean control [138]. However, these findings were recently challenged, demonstrating that Glut1 expression and glycolysis, assessed by extracellular acidification rate, in peritoneal macrophages are decreased in a mouse model of diabetes-accelerated atherosclerosis with low-density lipoprotein receptor deficiency [139]. However, whether these metabolic changes in macrophages are due to diabetes, atherosclerosis, or a combination of both is unclear. It is also important to emphasize that this was a mouse model of type 1 diabetes, not a T2D mouse model. Therefore, directly assessing the metabolic flux of glucose uptake and glycolysis rate in macrophages under T2D conditions will be important to clarify this controversy. In addition to stimulating glycolysis, an increase in glucose uptake in T2D can also lead to the formation of AGEs in macrophages. Studies have shown that AGEs activate the NF-κB signaling pathway to induce the production of pro-inflammatory cytokines. This activation is accompanied by increased production of inflammatory signaling factors, such as IL-1β and TNF-α [140].

#### Fatty acids

In addition to glucose, macrophages are sensitive to changes in circulating lipids levels. Augmented levels of circulating fatty acid act in T2D stimulate the proliferation of local macrophages residing within the adipose tissue [141]. In addition, fatty acids can also serve as a signaling molecule to activate pro-inflammatory signaling pathways, such as TLR4 [142]. It has been reported that M1-like macrophages with impaired oxidative phosphorylation could shift free fatty acids away from catabolism toward lipid synthesis, such as triglyceride [125]. Fatty acid synthesis is crucial for maintaining the phenotype and function of M1-like macrophages, as fatty acids are essential building blocks for cellular membranes and inflammatory mediators [94]. The role of fatty acid synthesis is discussed in detail in Sect. (“Metabolic regulation of M1-like macrophage”).

Macrophages in the setting of T2D are considered metabolically activated, as they mirror both the metabolic phenotypes of M1- and M2-like macrophages [118, 143]. Metabolically activated macrophages observed in the setting of T2D demonstrate increased glycolytic rates but also present with higher OXPHOS activity [144]. A study by Sharma et al. showed that both glycolytic rates and OXPHOS are elevated in adipose tissue macrophages in obese mice, and OXPHOS are elevated compared to lean controls. The study proposes that HIF-1α induces the production of IL-1β and targeted genes in the glycolytic pathway. Macrophages within adipose tissue especially are faced with accumulated lipids due to increased lipolysis of lipid storage at the state of insulin resistance [145]. Thus, lipids and fatty acids can be taken up by macrophages and catabolized through the lysosomal pathway as opposed to β-oxidation [146]. The production of inflammatory lipids, such as eicosanoids, is shown to be coupled with high levels of free fatty acids, which can further intensify insulin resistance in T2D [105, 147]. Increased lipid accumulation is present at the heart in the setting of diabetic cardiomyopathy, which can impair the function of microvascular networks, inducing capillary rarefaction [148]. This can lead to areas of the heart facing the condition of hypoxia. [149] HIF-1α can induce metabolic changes in macrophages away from oxidative phosphorylation toward enhanced glycolysis, which in turn polarizes the macrophages to an M1-like state with pro-inflammatory signatures [85]. However, stimulation of HIF1α can occur with nonhypoxic mechanisms [142]. Saturated fatty acid and oxidized low-density lipoprotein (LDL) can activate HIF-1α to polarize macrophages toward a more glycolytic phenotype with pro-inflammatory actions [150]. How HIF-1α influences macrophage metabolism and phenotype in T2D is yet to be directly investigated.

#### Ketones

In T2D, ketone bodies play a pivotal role in influencing macrophage activity via acting as signaling molecules. While ketone bodies generally promote an anti-inflammatory response, elevated levels in diabetic conditions may provoke a pro-inflammatory state. For instance, high circulating levels of the ketone body enhance TNF-α secretion in cultured U937 monocytic cells and circulating TNF-α levels in diabetic patients, effects that are mediated, at least in part, by ketone-induced cellular oxidative stress and cAMP deficiency [151]. Kanikarla-Marie et al. showed that ketone-induced increases in ROS, ICAM-1 expression, and monocyte adhesion are prevented in NADPH oxidase 4 (NOX4) knockdown cells [152], implicating NOX4 in mediating ketone-induced ROS generation. However, different ketone bodies might have divergent effects on inflammation and oxidative stress in T2D. A study by Kurepa et al. demonstrated that the monocyte chemotactic protein -1 (MCP-1) level correlates with circulating levels of AcAc, but not βOHB, in type 2 diabetic patients [153]. In further support of that, exposing cultured monocytes to high levels (4 mM) of AcAc, but not βOHB, increases MCP-1 secretion in vitro [153]. While these studies underscore the importance of ketone bodies in modulating macrophage phenotype in T2D as signaling molecules, it is still not fully understood how augmented ketone body levels in T2D influence macrophage energy metabolism in T2D.

#### Amino acids

**Fig. 1:**
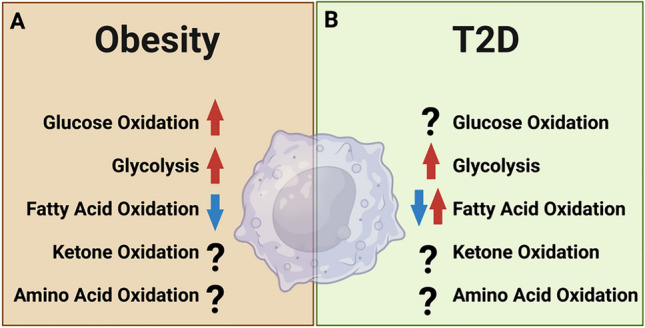
Summary of the alterations in macrophage energy metabolism in (**A**) obesity and (**B**) type 2 diabetes (T2D)

Elevated levels of branched-chain amino acids (BCAAs), namely leucine, isoleucine, and valine, correlate with the development of insulin resistance and predisposition to T2D [154]. BCAAs are shown to influence immune cell inflammatory responses, yet their exact impact still remains uncertain. In cultured human peripheral blood mononuclear cells (PBMCs), high levels (10 mM) of BCAA enhance ROS generation via both NADPH oxidase and the mitochondria. and stimulate Akt-mTOR signaling [155]. BCAAs also trigger the activity of the redox-sensitive transcription factor NF-κB, which results in the release of pro-inflammatory molecules, such as IL-6, TNF-α, ICAM-1, or CD40L, and the migration of PBMCs [155]. However, there is still controversy about whether BCAAs promote pro- or anti-inflammatory macrophages. For example, Lee et al. showed that high BCAA levels inhibit iNOS activity and reduce the expressions of IL-6 and COX-2 in LPS-stimulated RAW 264.7 macrophages [156]. Future research should clarify whether these effects occur through modulating macrophage energy metabolism as fuels or acting as signaling molecules in macrophages in the context of T2D (Fig. 1A, B).

### Atherosclerosis

Atherosclerosis is a progressive inflammatory disease that affects the artery [157]. It is characterized by an increased accumulation of lipids, cellular debris, and calcium within the arterial wall, forming plaques [157]. These plaques can obstruct the blood flow, which leads to serious cardiovascular complications, such as myocardial infarction and stroke [157]. Macrophages are directly involved in plaque formation and plaque rupture [158]. They are recruited to the lesion site to engulf and clear out the accumulated lipoproteins, such as LDL, but when excessive amounts of lipoproteins are present and exceed the capacity of macrophages, they transform into foam cells [159]. Of importance, studies have shown that the metabolism of macrophages undergo significant metabolic alterations in atherosclerosis [160].

#### Glucose

Studies have shown that oxidized LDL (Ox-LDL) promote atherosclerosis by enhancing macrophage foam cell formation and sterile inflammation [159, 161]. Macrophages treated with Ox-LDL accumulate modified lipids, accompanied by IL-1β production in humans and murine studies [162, 163]. Circulating monocytes from symptomatic atherosclerotic patients exhibit a pro-inflammatory phenotype and increased levels of glycolytic enzymes [89, 164, 165]. Pyruvate kinase muscle isozyme M2 (PKM2) is a rate-limiting glycolytic enzyme that facilitates the transfer of the phosphoryl group from phosphoenolpyruvate to ADP, thus generating ATP and pyruvate. Such stimulated glycolytic rates contribute to foam cell formation, as PKM2 also interact with sterol regulatory element-binding proteins (SREBP1), which upregulates the expression of lipogenic genes and increases macrophage cholesterol synthesis. SREBP1 also modulates lipoprotein uptake while suppressing protein expression related to cholesterol efflux through its effect on CD36 and FAS [165]. Collectively, this will lead to fatty acid and free cholesterol accumulating within the macrophages, stimulating foam cell formation. Accumulated free cholesterols also activate the TLR4, NF-κB, and NLRP3 signaling pathway to stimulate inflammatory activation of macrophages [166, 167].

#### Fatty acids

In atherosclerosis, macrophages can become foam cells when facing excessive LDL or oxidized LDL. This is primarily due to increased LDL uptake coupled with the impaired efflux of LDL. Although it is not fully defined how fatty acid oxidation might be altered, some indirect evidence suggests that macrophage fatty acid oxidation might be impaired in atherosclerosis [168]. For instance, Nomura et al. showed that genetic deletion of CPT1 or CPT2 in macrophages leads to impaired macrophage fatty acid oxidation [169]. This is accompanied by increased expression of CD36 for uptake of oxidized LDL and accelerated transformation of macrophages into foam cells [169]. CPT2 deletion also accelerates atherogenesis in ApoE knockout mice, with increased formation of foam cell macrophages and upregulated CD36 receptors at the aortic root [169]. This suggests the possibility of modulating macrophage fatty acid metabolism to interfere with plaque formation. In addition, intracellular lipids have also been shown to act as signaling molecules to regulate PPARα and liver X receptor α (LXRα) [170], where both contribute to the upregulation of genes involved in lipid uptake. Schneider et al. showed that the inactivation of FASN in macrophages of ApoE-deficient mice results in a decrease in diet-induced atherosclerosis and diminishes foam cell formation [170]. Therefore, it is likely that FAS can modulate the production of regulatory lipids that can transcriptionally control the formation of foam cells.

#### Ketones

Although there is no direct assessment of ketone oxidation flux in macrophages in atherosclerosis, some recent studies proposed that impaired ketone oxidation is observed in atherosclerosis [171, 172]. In patients with T2D and atherosclerosis, vascular expression levels of BDH1 are decreased [171], which is consistent with decreased BDH1 expression in the aorta from diabetes-induced atherosclerosis and ApoE^−/−^ mice models [171]. Interestingly, silencing macrophage BDH1 increases ROS generation and triggers inflammatory response in macrophages [171]. Mechanistically, BDH1 activates the Nrf2 pathway by regulating the metabolic flux of fumarate, which inhibits oxidative stress and leads to a decrease in ROS and inflammatory factor production in Raw264.7 macrophages in vitro [171]. Interestingly, overexpression of BDH1 attenuates aortic plaque formation and decreases inflammatory cytokine levels, such as IL-1β, IL-18, and TNF-α [171]. In another independent study, Zhang et al. demonstrated that daily oral administration of βOHB in ApoE^−/−^ mice reduces circulating levels of inflammatory cytokines, such as TNF-α, and suppresses plaque formation [172]. This beneficial effect is mediated by βOHB acting on receptor G-protein-coupled receptor 109a (Gpr109a) to inhibit NLRP3 inflammasome activation, which triggers M1-like macrophage proportion and inhibits cholesterol efflux via promote extracellular calcium influx and reducing endoplasmic reticulum stress [172]. These findings underscore the potential for modulating macrophage ketone metabolism to intervene in atherogenesis.

#### Amino acids

It has been shown that macrophage Arg-1 expression is upregulated in foam cell macrophages in human atherosclerotic plaques [24, 173]. Importantly, it is well established that pro-inflammatory cytokines such as IL-4 can upregulate the expression of Arg-1 [93, 174, 175]. In the context of atherosclerosis, a recent study has shown that upregulated expression of macrophage Arg-1 is crucial for resolving the accumulation of apoptotic cells by continual efferocytosis [24]. The study demonstrated that Arg-1 and ornithine decarboxylase are critical in metabolizing apoptotic cell-derived arginine and ornithine to putrescine in efferocytosis in vivo and atherosclerosis regression [24]. In addition, mice lacking Arg-1 showed defective efferocytosis and impaired lesion regression [24]. These studies suggest the pertinent role of amino acid metabolism in macrophages in mediating atherosclerosis resolution. Yet, the roles of other amino acids in modulating macrophage responses in atherosclerosis are unclear.

### Heart failure

Heart failure can be characterized into two main types: heart failure with reduced ejection fraction (HFrEF) and heart failure with preserved ejection fraction (HFpEF). Both the symptoms and the pathophysiology are very different. HFrEF mostly occurs, followed by myocardial infarction (MI), and is presented with impaired contractile function [176]. HFpEF has a very heterogeneous phenotype, is associated with many risk factors, including hypertension, obesity, T2D, aging, and chronic kidney disease, and is characterized by prominent diastolic dysfunction [177, 178]. Despite these differences in pathophysiology and manifestation, HFrEF and HFpEF are both associated with significant metabolic changes in the whole body as well as at the level of the heart [179–182]. Inflammation is a critical response in the heart under stress and can be viewed as an adaptive remodeling at the early stage [183]. However, if the stress persists and/or exacerbates, the inflammatory response might lead to cardiac injury. Within this process, macrophages are known to be actively involved. The activation of macrophages induces the secretion of pro-inflammatory cytokines, which are known to induce cardiac injury [184]. This is supported by the positive correlation between heart failure mortality and worse outcomes of patients with heart failure with the circulating levels of pro-inflammatory cytokines and monocytes [184].

#### HFrEF

In a healthy heart, resident cardiac macrophages account for 5%–10% of non-myocytes [185, 186] and present with M2 phenotypes [187, 188]. They retain cardiac homeostatic function by removing damaged cells, combating infection, and promoting tissue rebuilding after injury [189]. A recent study found that resident cardiac macrophages are attributed to electrical conduction [190]. In fact, the atrioventricular (AV) node is located with a large number of macrophages [190]. Removing resident cardiac macrophages impairs AV conduction and induces AV block [190]. While the role of macrophages is well recognized in heart failure, it is unclear how macrophage energy metabolism might be altered in different forms of heart failure and how that might influence macrophage phenotype and/or the progression of HFrEF and HFpEF.

##### Glucose

**Fig. 2:**
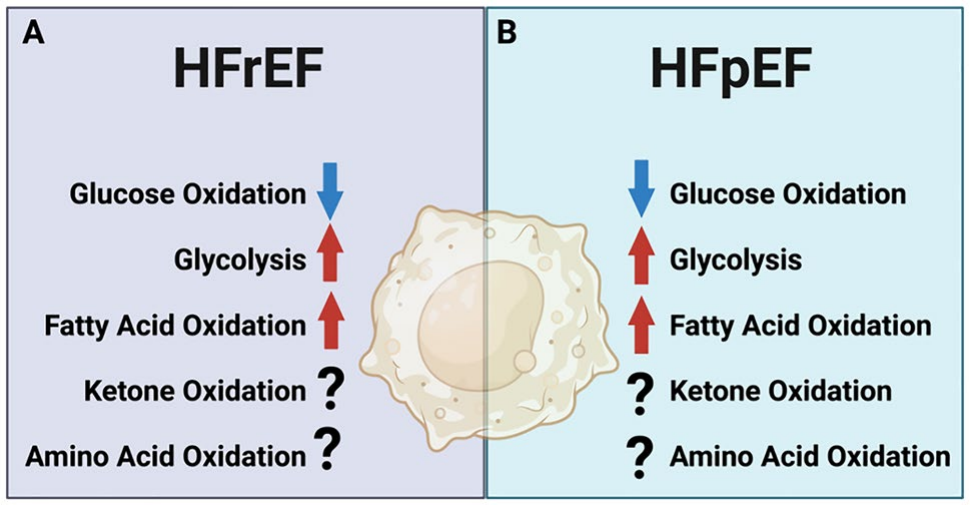
Summary of the alterations in macrophage energy metabolism in (**A**) heart failure with reduced ejection fraction (HFrEF) and (**B**) heart failure with preserved ejection fraction (HFpEF)

As occlusion of the coronary artery-induced MI can significantly alter the blood flow and oxygen supply to the heart, this can also lead to alterations in nutrient availability encountered by the heart and cardiac macrophages [191, 192]. Altered accessibility to extracellular glucose, fatty acids, amino acids, and possibly ketones can provide a rather challenging scenario for metabolic demanding macrophages [191]. Interestingly, the expression of metabolic enzymes is tightly correlated with the progression of MI [193]. Glycolytic genes such as GAPDH are shown to be significantly elevated in macrophages following MI [193]. This indicates the polarization toward M1-like phenotypes and the initiation of pro-inflammatory processes. Three days after MI, the genes involved in the TCA cycle, such as succinate dehydrogenase, are elevated [193]. This indicates that the macrophages are actively adapting to the changes in environments; when oxygen becomes more available, mitochondrial OXPHOS can be re-initiated in macrophages (Fig. 2A). Mouton et al. demonstrated that alterations in the metabolic profile of macrophages are temporal dependent in a mouse model of MI [194]. The study also showed that the phenotype of cardiac macrophages is dominated by M1 like on day one after MI and that was associated with the upregulation of several glycolytic genes, including GAPDH, LDHA, and PKM2 (Fig. 2A). Using the Seahorse assay, the authors demonstrated that glycolysis in macrophages is elevated from day 1 to 3 post-MI, and it returns to baseline on day 7. However, macrophage glucose oxidation rates are suppressed on days 1–3 post-MI and returned to baseline at seven-day post-MI, suggesting a mismatch between glycolysis and glucose oxidation in macrophage post-MI.

##### Fatty acids

The reparative action of efferocytosis to clean out apoptotic cells in infarct area post-MI is linked to the upregulation of fatty acid metabolism in macrophages [195]. Using unbiased global metabolic pathway analysis, Zhang et al. demonstrated that activation of cardiac macrophages by IL-10 following myocardial infarction is independent of glycolysis yet supported by apoptotic cell fatty acids and mitochondrial β-oxidation, the ETC, and heightened coenzyme NAD^+^ [195]. Catabolism of fatty acids is essential for producing anti-inflammatory cytokines, such as IL-10 [196]. The regulation of anti-inflammatory response by macrophages is also partly mediated by the level of NAD^+^ and its associated protein SIRT1 for transcriptional regulation [197]. However, how alterations in macrophage fatty acid oxidation might influence macrophage responses and phenotypes post-MI is still not fully understood.

##### Ketones

The literature regarding how ketones are metabolized in macrophages in the setting of HFrEF is rather limited. It has been shown that cardiac ketone oxidation is upregulated in HFrEF in both preclinical [198, 199] and clinical settings [200]. Investigating whether macrophage ketone metabolism is also altered in HFrEF or whether the alterations in ketone metabolism in cardiac macrophages contribute to the progression of adverse remodeling and cardiac dysfunction would be an interesting research scope for future studies.

##### Amino acids

Glutamine, the most abundant amino acid in plasma, can be metabolized by macrophages [201]. Merlin et al. reported that glutaminase-1-mediated glutaminolysis is important in stimulating apoptotic cell clearance by macrophages [202]. The study also proposed that the non-canonical transaminase pathway is crucial for supporting macrophage’s efferocytotic capacity, which is independent of the canonical glutamine catabolism pathway that leads to the generation of α-KG into the TCA cycle through the action of glutamate dehydrogenase. However, whether the glutaminase-1 activity is upregulated in HFrEF or how manipulating glutaminolysis influences macrophage energy metabolism and phenotype in HFrEF is unclear.

Succinate is linked to macrophage activation through the activity of succinate dehydrogenase [89]. Of importance is that succinate can be shuttled from mitochondria to the cytosol, where it modulates prolyl hydroxylase activity, causing HIF-1α stabilization and pro-inflammatory IL-1β production [85]. Succinate accumulation is also shown to activate the NLRP3 inflammasome and trigger mitochondrial ROS generation, both of which can exacerbate myocardial ischemia–reperfusion [203]. Another alteration in macrophage metabolism that leads to succinate accumulation occurs at the level of isocitrate dehydrogenase [77]. Decreased isocitrate dehydrogenase activity leads to the accumulation of itaconate [84], a metabolic signature of activated macrophages [204], which inhibits succinate oxidation.

α-KG, produced by the TCA (or generated from glutaminolysis), is an important co-factor for several enzymes involved in epigenetic modifications in macrophages [205]. α-KG accumulates in alternatively activated macrophages, whereas its abundance is decreased in classically activated macrophages due to higher α-KG dehydrogenase activity [84]. Of interest is that the α-KG/succinate ratio in macrophages can modulate Jmjd3 activity [96]. Jmjd3, an essential H3K27 demethylase, contributes to the activation of alternative macrophages, whereas its activity attenuates inflammation in classically activated macrophages. Thus, manipulating this ratio may influence the macrophage polarization and capacity for tissue repair in HFrEF.

#### HFpEF

Macrophages play various roles in adverse cardiac remodeling and diastolic dysfunction in HFpEF by different mechanisms. It has been shown that the quantity of cardiac macrophages is doubled in abundance in myocardial biopsies from patients with HFpEF [206]. Epicardial adipose tissue is a great reservoir of macrophages, as macrophages are one of the most abundant immune cells within adipose tissue. Of importance, studies have shown that obese patients with HFpEF have increased thickening of epicardial adipose tissue [177, 207]. This might suggest that this increase in adipocyte size causes local hypoxia and induces HIF-1α activation and polarization of macrophages in the epicardial adipose tissue toward the M1-like phenotype. However, this is yet to be directly assessed. In healthy conditions, most macrophages within the epicardial adipose tissue are M2 like and are responsible for protecting the heart from infection [121]. Alternatively, in facing stresses, such as coronary artery disease, macrophages within the epicardial adipose tissue can be polarized to M1 phenotypes, promoting pro-inflammatory processes and adverse remodeling of the cardiomyocytes [121].

##### Glucose

The current literature regarding macrophage metabolism in HFpEF is rather limited. However, some information can be obtained from looking at the alterations in macrophage metabolism in some of the main co-morbidities associated with HFpEF. For instance, Min et al. showed that M1-like macrophage polarization is impaired in peritoneal adipose tissue in obese mice [208]. This was associated with upregulation of both PDK2 and PDK4, suggesting decreased macrophage glucose oxidation (Fig. 2B). Of importance is that deletion of both PDK2 and PDK4 is associated with reduced weight gain, insulin resistance, and adipose tissue inflammation, emphasizing the promising effect of targeting macrophage glucose oxidation in mitigating insulin resistance and tissue inflammation in obesity.

##### Fatty acids

A recent study from Liu et al. showed an increased cardiac IL-1β level in a high-fat diet-induced HFpEF mouse model [209]. The source of the IL-1β was attributed to the increased infiltration of inflammatory M2-like macrophages. Interestingly, deletion of fatty acid-binding protein 4 (FABP4, an intracellular lipid chaperone highly expressed in adipocytes) abolished the ability of macrophages to release IL-1β in response to LPS stimulation and improved diastolic function (E/e’ ratio) in HFpEF mice. These findings suggest that fatty acid metabolism is crucial for regulating the production of pro-inflammatory cytokines and maintaining the pro-reparative M2-like macrophages in HFpEF [195].

##### Ketones

Youm et al. reported that βOHB has signaling properties, and it blocks the NLRP3 inflammasome in macrophages and IL-1β and IL-18 production, suggesting the anti-inflammatory property of βOHB [210]. Another study by Taggart et al. demonstrated that βOHB is an endogenous ligand for the hydroxycarboxylic acid receptors HCA2, which is highly expressed by immune cells, including macrophages [211]. HCA2 has been suggested to mediate profound anti-inflammatory effects [212]. Therefore, βOHB might also exert anti-inflammatory effects via acting on HCA2 and suppressing activation of NLRP3 inflammasome. However, the role of macrophage ketone metabolism in modulating macrophage phenotype in HFpEF remains unknown.

## Concluding remarks

Macrophages are a diverse group of cells that can promote inflammation and phagocytosis of extracellular debris. They are present in different tissues and contribute to maintaining tissue health by responding to various molecules in their surrounding environment through cell surface receptors. Recent studies have linked macrophage metabolic processes to their inflammatory behavior and demonstrated that macrophages could switch from promoting tissue protection to contributing to disease development. Although macrophage insulin signaling is impaired in cardiometabolic disease (obesity and T2D, atherosclerosis, and heart failure), insulin-resistant macrophages have increased glucose uptake, glycolysis, and glucose oxidation. Macrophage fatty acid uptake is also increased, but it seems uncoupled to fatty acid oxidation, which is decreased in obesity and T2D. Instead, fatty acids are converted to triacylglycerol and ceramide accumulation, contributing to insulin-resistant macrophages’ lipotoxicity. Arginine and glutamate metabolism also have divergent effects in pro- and anti-inflammatory macrophages in obesity and T2D. Macrophage fatty acid oxidation is upregulated in heart failure. However, macrophage glucose oxidation is impaired and uncoupled from high glycolytic rates in macrophages. Understanding the complex metabolic profile of different macrophage phenotypes will help characterize how different oxidative substrates could influence macrophage responses. In addition, understanding the metabolic reprogramming behind macrophage responses will help identify new avenues for therapeutic intervention to combat cardiometabolic diseases.

## Data Availability

No datasets were generated or analysed during the current study.
