# Correction to: Macrophage energy metabolism in cardiometabolic disease

**DOI:** 10.1007/s11010-024-05197-5

**Published:** 2025-01-23

**Authors:** Angela Wong, Qiuyu Sun, Ismail I. Latif, Qutuba G. Karwi

**Affiliations:** 1 Division of BioMedical Sciences, Faculty of Medicine, Memorial University of Newfoundland, St. John’s, Newfoundland and Labrador A1B 3V6 Canada; 2 Cardiovascular Research Centre, University of Alberta, Edmonton, Alberta Canada; 3 Department of Microbiology, College of Medicine, University of Diyala, Baqubaa, Diyala Iraq

**Correction to: Molecular and Cellular Biochemistry** 10.1007/s11010-024-05099-6

The article “Macrophage energy metabolism in cardiometabolic disease”, written by Angela Wong, Qiuyu Sun, Ismail I. Latif and Qutuba G. Karwi was originally published Online First without Open Access. With the author(s)’ decision to opt for Open Choice the copyright of the article changed on 18th December 2024 to © The Author(s) 2024 and the article is forthwith distributed under a Creative Commons Attribution 4.0 International License, which permits use, sharing, adaptation, distribution and reproduction in any medium or format, as long as you give appropriate credit to the original author(s) and the source, provide a link to the Creative Commons licence, and indicate if changes were made. The images or other third party material in this article are included in the article’s Creative Commons licence, unless indicated otherwise in a credit line to the material. If material is not included in the article’s Creative Commons licence and your intended use is not permitted by statutory regulation or exceeds the permitted use, you will need to obtain permission directly from the copyright holder. To view a copy of this licence, visit http://creativecommons.org/licenses/by/4.0/.

The original article has been corrected.

